# De La Circulation Du Sang Dans Les Membres Et Dans La Tête Chez L’Homme

**Published:** 1860-09

**Authors:** 


ART. XII.—*De la Circulation du Sang dans les Membres et dans la Tête chez l'Homme.* Par J. P. SUCQUET, Docteur en Médecine de la Faculté de Paris; Lauréat de l'Académie des Sciences; Chevalier de la Légion d'Honneur. 8vo., pp. 55. Paris: J. B. Baillière et Fils, 1860.

THE experiments and observations embodied in this little pamphlet are of a highly interesting character, and if verified by subsequent investigation, will, to a certain extent, modify our views both of the physiology and pathology of the circulation. By throwing solidifiable injections into the arteries, and subsequently dissecting and carefully tracing out the vessels under a lens, M. Sucquet claims to have discovered, in the upper and lower extremities, and the head, a peculiar, derivative, or diverticular circulation. He maintains that in each of these parts there is, independent of the capillary system, a direct passage between certain arteries and veins. He injected the axillary artery and found that the veins of the fingers and hand were the first to swell with the injected liquid, then the subcutaneous trunks of the forearm, and finally those of the arm. The trunks of the cephalic and basilic veins received the injection freely from the veins of the hand and fingers. Those branches of these two veins, however, which originate in the skin of the forearm, and the muscular and other deep-seated veins, were empty and collapsed. It is generally supposed that in the arm the blood passes from the arteries into the veins everywhere in the same manner. This our author denies. He contends that there are two distinct circulations in the upper extremity. One of these is general, permanent, regular, and nutritive; the other is inconstant and irregular, and has nothing to do with nutrition. It is localized in certain vessels of the elbow, and, above all, in the arteries and veins of the hand, and acts as a diverticulm for the superabundant blood of the arterial system of the arm, counteracting the frequent and marked irregularities of the latter, and preserving the constancy and the equality of the interstitial circulation.

M. Sucquet has also studied the circulation in the lower extremities. He found that liquids thrown into the crural artery always returned by the crural and great saphena veins; more quickly and more abundantly by the former, however, than by the latter. His observations led him to conclude that the lower extremity, like the upper, possesses not only a general circulation which flows through capillary vessels and is constant, equable, and nutritive, but another or supplementary circulation which is irregular and derivative, is confined to certain vessels of the knee, and especially to particular arteries of the foot, anastomosing with the saphena and even with the deep-seated veins at the end of the limb.

Injections cast into the primitive carotid artery always return, according to our author, by the jugular veins of the neck, without having visibly distended the vessels of the head. The derivative circulation of this part of the body is seated in the face, being carried on by the facial, ophthalmic, and auricular vessels. The surplus blood of the arterial system is transferred to the venous chiefly by the facial vein.

The derivative circulation, wherever it occurs, is particularly under the influence of the heart. Whatever increases the strength and rapidity of the latter, augments also the activity of the former. The ingestion of food, alcoholic drinks, joy, anger, violent exercise, heat, and certain febrile movements accelerate this circulation by hurrying the action of the heart. On the other hand, hunger, fright, the depressing passions, and cold lessen this circulation by weakening the heart.

We regret that our limits forbid a more extended notice of the novel and important views of M. Sucquet. Those who are interested in these anatomico-physiological inquiries will be amply repaid by an attentive perusal of his very suggestive pamphlet.

